# *In vivo* Measurements of Electric Fields During Cranial Electrical Stimulation in the Human Brain

**DOI:** 10.3389/fnhum.2022.829745

**Published:** 2022-02-18

**Authors:** Minmin Wang, Tao Feng, Hongjie Jiang, Junming Zhu, Wuwei Feng, Pratik Y. Chhatbar, Jianmin Zhang, Shaomin Zhang

**Affiliations:** ^1^Key Laboratory of Biomedical Engineering of Education Ministry, Zhejiang Provincial Key Laboratory of Cardio-Cerebral Vascular Detection Technology and Medicinal Effectiveness Appraisal, Department of Biomedical Engineering, School of Biomedical Engineering and Instrument Science, Zhejiang University, Hangzhou, China; ^2^Department of Neurosurgery, The Second Affiliated Hospital, Zhejiang University School of Medicine, Hangzhou, China; ^3^Department of Neurology, Duke University School of Medicine, Durham, NC, United States; ^4^Qiushi Academy for Advanced Studies, Zhejiang University, Hangzhou, China

**Keywords:** cranial electrical stimulation, *in vivo*, modeling, validation, sEEG

## Abstract

Cranial electrical stimulation (CES) has been applied at various current levels in both adults and children with neurological conditions with seemingly promising but somewhat inconsistent results. Stimulation-induced spatial electric fields (EFs) within a specific brain region are likely a significant contributing factor for the biological effects. Although several simulation models have been used to predict EF distributions in the brain, these models actually have not been validated by *in vivo* CES-induced EF measurements in the live human brain. This study directly measured the CES-induced voltage changes with implanted stereotactic-electroencephalographic (sEEG) electrodes in twenty-one epilepsy participants (16 adults and 5 children) and then compared these measured values with the simulated ones obtained from the personalized models. In addition, we further investigated the influence of stimulation frequency, intensity, electrode montage and age on EFs in parts of participants. We found both measured voltages and EFs obtained *in vivo* are highly correlated with the predicted ones in our cohort (Voltages: *r* = 0.93, *p* < 0.001; EFs: *r* = 0.73, *p* < 0.001). In white matter and gray matter, the measured voltages linearly increased when the stimulation intensity increased from 5 to 500 μA but showed no significant changes (averaged coefficient of variation <4.10%) with changing stimulation frequency from 0.5 to 200 Hz. Electrode montage, but not age, significantly affects the distribution of the EFs (*n* = 5, *p* < 0.01). Our *in vivo* measurements demonstrate that the individualized simulation model can reliably predict the CES-induced EFs in both adults and children. It also confirms that the CES-induced EFs highly depend on the electrode montages and individual anatomical features.

## Introduction

Cranial electrical stimulation (CES) is a form of transcranial electrical stimulation (tES) which modulates neural activity with pulse current delivered *via* surface electrodes (Jonas, 2018). It is distinguished from the other forms of tES, such as transcranial direct current stimulation (tDCS) or transcranial alternating current stimulation (tACS), by the variation in electrode montage and stimulation waveform. Generally, CES frequencies and intensities range from 0.5 to 100 Hz and from 50 μA to 4 mA, respectively. Ear lobes, mastoid process, temple, infra-auricular and supra-auricular are commonly used electrode positioning sites for CES (Brunyé et al., 2021). With the practical advantage of flexibility, low cost, and good tolerance, CES has been investigated for treating anxiety (Morriss and Price, 2020), depression (McClure et al., 2015), chronic pain (Tan et al., 2006), insomnia (Wagenseil et al., 2018). However, the underlying mechanisms of CES still remain controversial. The CES-induced spatial electric fields (EFs) within the corresponding brain regions are likely a significant factor for the biological effects of CES. Several studies have provided evidence for the effects of EFs on neural activity (Sun et al., 2016; Kronberg et al., 2020; Farahani et al., 2021). However, it is extremely difficult to obtain the EF distributions directly from live human brains during the stimulation process.

As an alternative way, simulation models have been developed to understand the spatial EF distributions throughout the whole brain (Ferdjallah et al., 1996). Datta et al. (2013) reported a simulation model of CES using elaborated structural magnetic resonance imaging (MRI) data. They found that significant amounts of current can penetrate the skull and reach cortical and subcortical structures (Datta et al., 2013). In addition, their results also showed that the electrode montage could significantly influence current flow distribution within the human brain. Although the simulation model provides a useful visualization technique to obtain the spatial EF distributions, it is worth noting that the clinical effects from CES are inconsistent yet. Significant inter-individual variability has been noted in many studies (Brunyé et al., 2021; Price et al., 2021). This could be due to multiple factors. For example, various stimulation parameters (e.g., current intensity, frequency, waveform, electrode placement, electrode size, number of treatment sessions, and stimulation duration) were adopted in different studies. On the subject level, variations in head geometry, skull thickness, brain anatomical difference, and tissues electrical characteristics are known to exist across subjects, especially between children and adults. Concerns have been raised about whether the existing simulation models take into account the effects from stimulus parameters and individual differences. Sufficient direct evidence is still lacking to validate these models’ prediction accuracy.

Electric fields data from *in vivo* measurements is crucial for the evaluation and exploration of CES. In a previous report (Dymond et al., 1975), the CES-induced voltage differences were measured using implanted electrodes in three epilepsy participants. However, the spatial EF distribution was not fully obtained due to the limitation of contemporary technical methods. Until recent years, some studies reported *in vivo* measurements of tES (Opitz et al., 2016; Lafon et al., 2017; Vöröslakos et al., 2018). Huang et al. (2017) performed model validation of tES by combining stereotactic-electroencephalographic (sEEG) and electrocorticographic (ECoG) measurements in epilepsy participants, but their results may be affected by skull defects from the surgically implanted ECoG electrode (Datta et al., 2010). In another study, Chhatbar et al. (2018) reported the measurement of tDCS-induced voltage by using deep brain stimulation (DBS) electrodes in three human participants with Parkinson’s disease. However, their data sampling is limited to a single or two electrodes. Collectively, there are only a handful of *in vivo* measurements, especially for different age groups. It is remarkable that the actual spatial EF distributions remain largely unclear during CES, which affected clinical application for CES to some extent.

In this study, with the advantage of minimally invasive used for sEEG, we aim to directly measure the CES-induced voltage changes with implanted sEEG electrodes in participants with drug-resistant epilepsy and to compare these measured values with computational ones obtained from the numerical simulation models based on their own individual MRI features. Furthermore, we investigate the influence of stimulation frequency, intensity, electrode montage and age on the EFs.

## Materials and Methods

### Participants

Twenty-one right-handed participants with epilepsy [8 females, 23.6 ± 9.2 years (range 7–37)] were recruited. Participants were divided into two groups, the adults group (*n* = 16, 5 females, 27.6 ± 6.3 years) and the children group (*n* = 5, 3 females, 11 ± 2.9 years). All participants were consented and recruited based on the following selection criteria. Inclusion criteria are: (1) undergoing intracranial recording to localize sites of seizure initiation for epilepsy surgery; (2) cognitive ability to complete questionnaires for adults; and (3) capable of providing informed consent; for children, informed consents were signed by their parents. Exclusion criteria are: (1) cognitive impairment (IQ < 70); (2) documented severe depression or other neuropsychiatric diseases; (3) scalp/skin disease; (4) frequent electro-clinical seizures within 24 h immediately after electrode implantation; and (5) space-occupying intracranial lesion. All procedures were performed following the Declaration of Helsinki. The Institutional Review Board at the Second Affiliated Hospital Zhejiang University School of Medicine approved the protocol. All participants (or legally authorized representatives) signed written consents before participating in the study.

### Cranial Electrical Stimulation Protocol

Because the linear and quasi-static assumptions were adopted in CES modeling, the simulated peak EF values were equal for tACS or CES at the same stimulation intensity. Considering the band-pass characteristics of clinical EEG amplifier, the transcranial sinusoidal alternating current stimulation were applied using a current stimulation device (Starstim 8, Neuroelectrics, Barcelona, Spain) in our study. In order to get high-quality recording data, circular Ag/AgCl electrodes (electrode area of 3.14 cm^2^) with conductive gel were used. As shown in Figure 5A, two stimulation electrodes were placed on the left and right pre-auricular or infra-auricular, respectively. Various stimulation intensities (5, 10, 25, 50, 100, 250, and 500 μA) and frequencies (0.5, 10, 50, 75, 100, 125, 175, and 200 Hz) were applied for some participants. Due to the restrains in our clinical trials, not all participants receive all stimulation studies. The specific electrical stimulation pattern for each participant were listed in the Supplementary Table I. Unless otherwise specified, recording values being analyzed were performed at 100 Hz/500 μA stimulation, and two CES electrodes were placed at left and right infra-auricular. For safety and tolerance, the intensities were capped at 250 μA for 5 children. Thus the measured voltage values at 250 μA stimulation were calibrated to correspond to 500 μA stimulation for children (the measured voltage at 250 μA stimulation were multiplied by 2). Each stimulation trial lasted for 180 s, including 30 s at baseline, 10 s for ramp-up, 100 s of stimulation time, 10 s of ramp-down and 30 s after stimulation. The same stimulation trial was repeated three times in one session. The mean measured value from three repeated stimulation trials was used for EF distribution estimation. The impedance at the electrode contacts was monitored during the stimulation period for safety purposes. The CES experiment was conducted within 2–10 days after sEEG implantation. A physician was present at the bedside during the entire study procedure to ensure safety. A detailed physical examination was performed before and after the experiment.

### Intracranial Electric Fields Recording Protocol

Cranial electrical stimulation induced intracranial voltages were recorded with clinically implanted sEEG electrodes (Sinovation, China; 0.8 mm diameter, 3.5 mm contact spacing, 2 mm contact length) that could access the depth of sulci and cortices on medial and basal surfaces of the cerebral hemispheres. The implantation sites and the number of the sEEG electrodes were determined by clinical considerations. A clinical amplifier (EEG-1200C, Nihon Kohden, Tokyo, Japan) was used to collect band-pass voltages filtered from 0.16 to 300 Hz and the sampling rate at 2,000 Hz. Due to clinical limitations, a clinical amplifier (Nicolet EEG, Natus Neuro, Middleton, MA, United States; 0.05–300 Hz band width, 2,000 Hz sampling rate) was adopted for participants S1 and S5. The common reference for the electrophysiological recordings was the average value of two clinically selected adjacent recording electrodes.

As shown in Supplementary Figure 1, the CES-induced voltage recordings were band-pass filtered in a narrow band around the applied stimulation frequency using a zero phase, second-order Butterworth IIR filter to reduce the noise interference. The magnitudes of CES-induced voltages were measured by fitting a sinusoid to the recordings during sinusoidal stimulation. The projected EF was calculated in the direction of adjacent recording electrodes by subtracting voltage values and dividing by their distance. We estimated the EF component along with the direction of measurement electrodes (projection of the EF). EF measurements were sensitive in the radial component (inward). Due to the noise interference, data from several electrodes could not be used in analysis. As a result, 2,587 of the 2,663 electrodes were included in the final data analysis: S1 (145/158), S2 (79/86), S3 (115/120), S4 (98/100), S5 (127/127), S6 (47/48), S7 (131/140), S8 (137/138), S9 (177/184), S10 (136/140), S11 (150/150), S12 (86/86), S13 (116/116), S14 (110/110), S15 (125/126), S16 (102/110), S17 (125/126), S18 (201/202), S19 (142/146), S20 (118/122), and S21 (120/128).

### Simulation Model and Data Analysis

**FIGURE 1:**
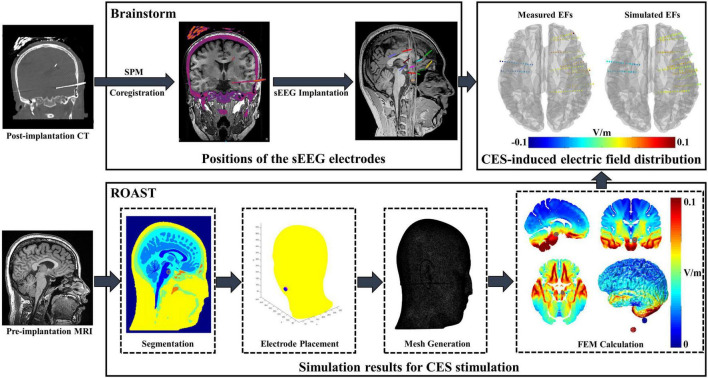
Electric field (EF) modeling and *in vivo* data analysis procedure. Brainstorm software was applied for individual post-implantation CT/pre-implantation MRI image registration. After manually editing the sEEG electrode site on the original CT, the positions of the electrode were exported in the MNI coordinates. ROAST was adopted to perform EF modeling by using individual MRI image. The voxel coordinates of the stimulation electrode were imported to ROAST, and then the stimulation electrodes were modeled at the customized locations. Combining with the simulated results and MNI coordinates of sEEG electrode, the voltage values of each electrode site were obtained and further compared with the measured value.

To compare the difference between simulated and actual measured CES-induced EF distributions, individual T1-weighted MRI images (for modeling) and CT images (for electrode positions acquisition) were acquired using a 3T MRI scanner (UIH Umr 790 system, TR = 8.2 ms, TE = 3.2 ms, flip angle = 12°) and CT scanner (SIEMENS SOMATOM Perspective, 237 mA/slice, 120 kV) with a resolution of 0.5 mm × 0.5 mm × 1.0 mm. As shown in Figure 1, we used Brainstorm software (brainstorm3)^1^ (Tadel et al., 2011) to co-register MRI and CT images for determining the precise locations of sEEG electrodes. Open-source software package ROAST (Realistic vOlumetric-Approach to Simulate Transcranial electric simulation, ROAST 3.0)^2^ (Huang et al., 2019) was adopted to perform EF modeling for CES. The resampled T1 images (1.0 mm × 1.0 mm × 1.0 mm) were individually processed in parallel using ROAST. ROAST called SPM12 to segment the MRI into gray matter (GM), white matter (WM), cerebrospinal fluid (CSF), bone, scalp and air cavities. Stimulation electrodes were modeled and placed at the customized locations on the scalp. Because the sEEG implantation was minimally invasive, the sEEG electrodes were not modeled in our simulation model. The volumetric mesh was created from 3D multi-domain images by using iso2mesh (Tran et al., 2020). Due to lack of diffusion tensor imaging (DTI) data, anisotropic tissue conductivity was not considered in this study. The conductivities of all tissues were assumed to be isotropic (WM: 0.126 S/m, GM: 0.276 S/m, CSF: 1.65 S/m, skull: 0.01 S/m, scalp: 0.465 S/m, air cavities: 2.5 × 10^–14^ S/m, electrode: 5.9 × 10^7^ S/m, gel: 0.3 S/m). The simulation model was solved to predict EF distributions by using a finite element solver, getDP. Pearson correlation coefficient was used for correlation analyses between the predicted values and the measured ones. A best-fit line was obtained by linear regression to evaluate the accuracy of the voltage or the EF distributions estimated by the simulation model.

## Results

### Participant’ Clinicodemographic Characteristics

**FIGURE 2:**
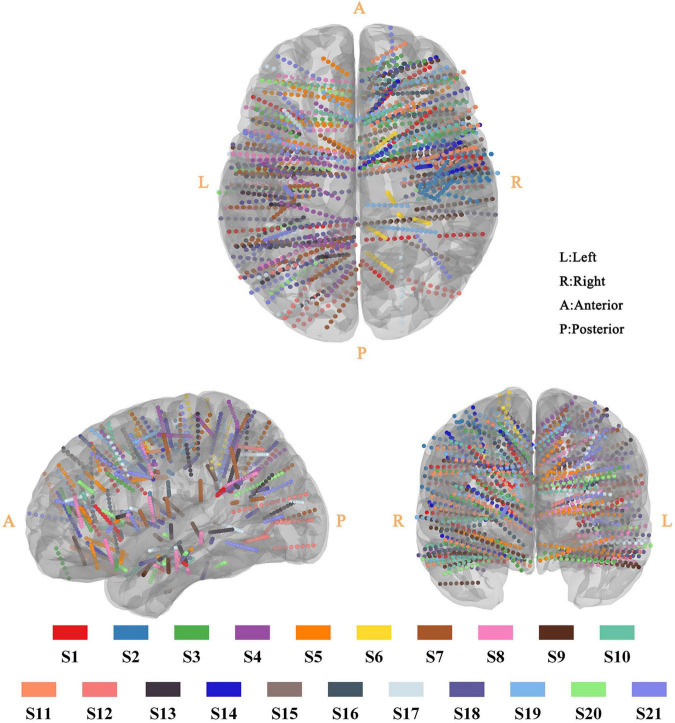
Three-dimensional reconstruction of sEEG electrode for all participant. All sEEG electrodes were normalized and reconstructed in the standard MNI-152 template. The sEEG electrodes from different participants were distinguished by the color-coding of participants at the bottom of the figure.

Twenty-one epilepsy participants (16 adults and 5 children) were recruited, and intracranial voltage changes were recorded with 2,587 sEEG recording electrodes in total. As displayed in Figure 2, these sEEG electrodes covered the subcortical brain area, extensive portions of the lateral and medial frontal, parietal and temporal cortex of the left and/or right hemisphere. The recording electrode locations were also presented as videos (Supplementary Video 1). Our study has a larger sample size, wider electrode coverage and more sEEG electrodes compared with previous *in vivo* studies (Huang et al., 2017; Opitz et al., 2018; Louviot et al., 2022).

### Measured and Simulated Electric Field

**FIGURE 3:**
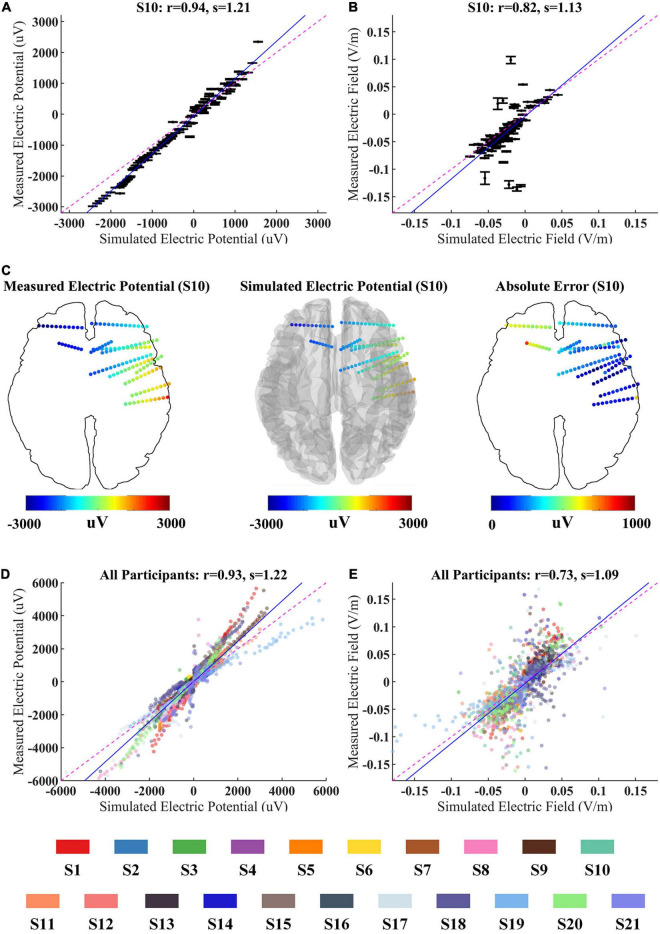
Correlation between simulated and measured values for 100 Hz/500 μA stimulation. **(A,B)** The measured voltages and projected electric fields (EFs) with simulated values from individualized model for S10. Error bars at each electrode indicate the variability across three repeated stimulation trials. **(C)** The spatial electric potential distributions from recorded and simulated results for S10. **(D,E)** Comparison of recorded voltages and projected EFs with predicted values for all participants. The data from different participant were represented by the color-coding of participants at the bottom of the figure. Points falling on the magenta line represent perfect prediction (slope *s* = 1). Blue line represents fitting line.

To validate whether the computational model can accurately predict the EF induced by CES, we first compared the differences between the measured and predicted fields. As shown in Figure 3A, the recorded voltages are highly correlated with the simulated voltage values for S10 (the Pearson correlation coefficient *r* = 0.94, *p* < 0.001; the slope of the best linear fit *s* = 1.21). Similar results are observed for all twenty-one participants (*r* = 0.93, *p* < 0.001; *s* = 1.22, Figure 3D). The spatial electric potential distributions from recorded and simulated results for S10 are displayed in Figure 3C. As shown in Figure 3B, we also found a high correlation between the measured and simulated values in the EF distributions. The Pearson correlation coefficient is *r* = 0.82, *p* < 0.001 for S10, which indicates that the EF could be predicted well by the simulation models. For all participants, the correlation of measured projected EFs and simulated ones decreased little (*r* = 0.73, *p* < 0.001, Figure 3E). We found that the correlation between measured projected EFs and simulated ones was lower than that of the voltage value, consistent with a previous study (Huang et al., 2017). The correlation coefficient and slope of the best linear fit between the measured and simulated values for each participant were illustrated in Supplementary Table II (for voltages: *r* = 0.94 ± 0.05, *s* = 1.32 ± 0.33; for projected electric fields: *r* = 0.71 ± 0.12, *s* = 1.25 ± 0.35). Overall, the results from *in vivo* measurement suggested the computational model could perform well for predicting the current distribution of CES.

### Intensity and Frequency Effects

As the stimulation parameters are likely essential for biological effects, we also investigated the impact of various combinations of intensity and frequency on EFs. CES was applied to these Six participants (S1–S5 and S8) through two electrodes placed at the left and right infra-auricular.

**FIGURE 4:**
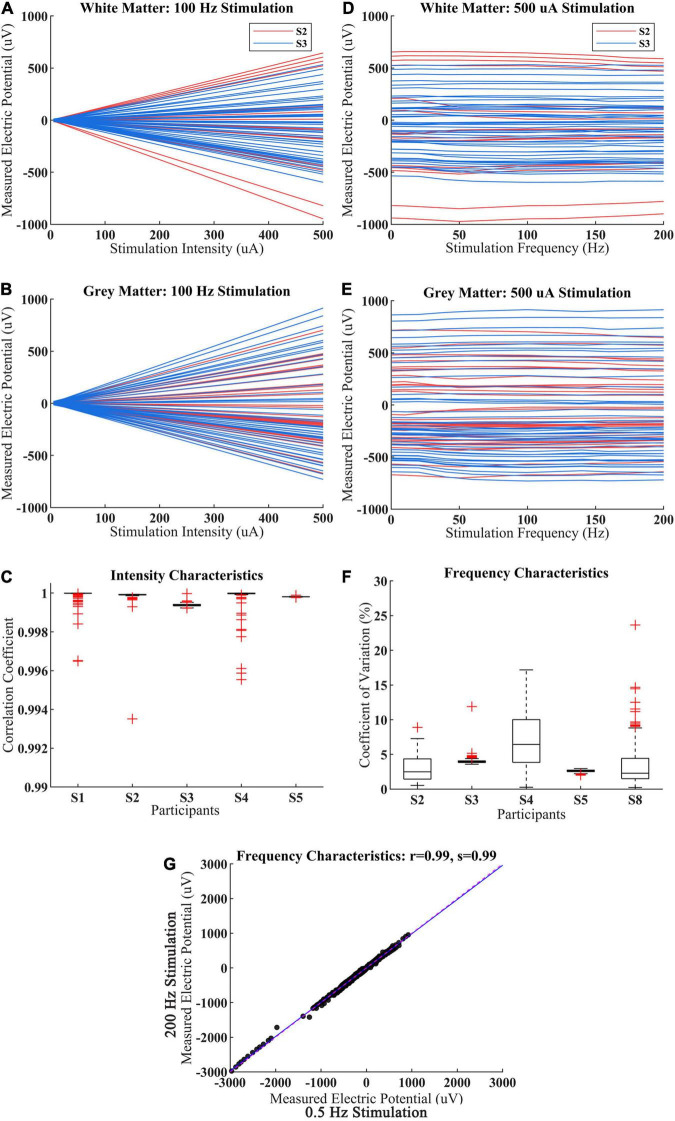
Intracranial voltage recordings for various stimulation intensity and frequency. **(A,B)** The measured voltage with stimulation intensity increase from 5 to 500 μA for the child (S2) and adult (S3) within WM and GM. Each red line represents a different electrode from S2, each blue line represents a different electrode from S3. **(C)** Under 100 Hz CES, the correlation coefficient of measured voltage with different stimulation intensity among participant S1–S5, the measured voltage derived from all sEEG electrode of each participant. **(D,E)** The measured voltage with stimulation frequency increase from 0.5 to 200 Hz for the child (S2) and adult (S3). Each red line represents a different electrode from S2, each blue line represents a different electrode from S3. **(F)** Under 0.5 mA CES, the coefficient of variation of measured voltages from different stimulation frequency among different participant S2–S5 and S8. **(G)** The measured voltage distributions between 0.5 and 200 Hz stimulation for participant S2–S5 and S8.

The magnitude of measured electric potential increased linearly with stimulation intensity from 5 to 500 μA in WM and GM for both adult and child participants (Figures 4A,B). The correlation coefficient of measured voltage and the stimulation intensity was very close to one among the five participants (S1–S5), which showed that the CES-induced potential changes have a good linearity with current magnitude in WM and GM (Figure 4C).

The measured voltages were consistent across CES stimulation frequency from 0.5 to 200 Hz in most recording electrodes from WM and GM (Figures 4D,E). Meanwhile, the averaged coefficient of variation of CES-induced voltage at different stimulation frequencies was less than 4.10% (Figure 4F), which indicated that the measured voltages changed minimally when the CES stimulation frequency increased from 0.5 to 200 Hz. There was also a slight inter-individual difference in frequency characteristics. We observed that the measured voltages induced by 0.5 Hz CES are almost the same with those values induced by 200 Hz stimulation across all electrodes among 5 participants in Figure 4G (the Pearson correlation coefficient *r* = 0.99, *p* < 0.001; the slope of the linear fit *s* = 0.99).

### Montage Effects

**FIGURE 5:**
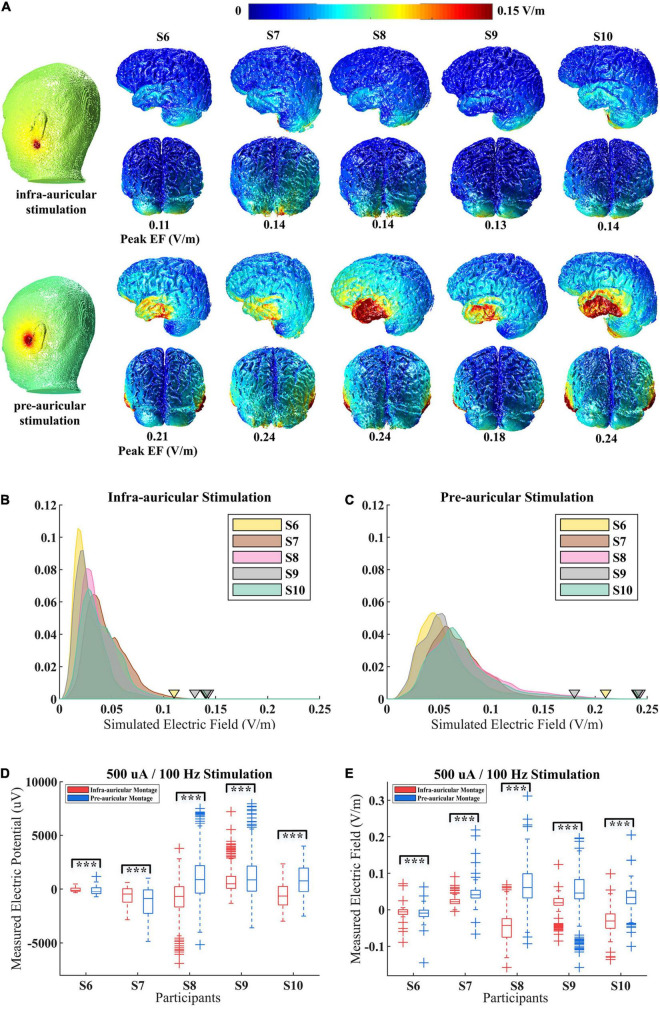
Simulated and measured electric field (EF) distributions for different electrode montage during CES. **(A)** Simulated EF distributions for S6–S10 with infra-auricular and pre-auricular stimulation. **(B,C)** The probability density distribution of EF within brain for infra-auricular and pre-auricular stimulation. The inverted triangle represents the peak EF. **(D,E)** Measured voltage and projected EF distributions for S6–S10 with infra-auricular and pre-auricular stimulation. ***p < 0.01.

As a main stimulation parameter of CES, many electrode montages have been applied. However, it remains unclear how the electrode montage affects actual spatial EFs distribution. To compare the effects of different electrode montages, two types of electrode montage (infra-auricular and pre-auricular stimulation) were used on participants (S6–S10). The simulation results show a significant difference in peak EF values for these two CES electrode placements (Figure 5A). The peak value of EF (i.e., the 99.9th percentiles) induced by the pre-auricular montage was much stronger than that induced by the infra-auricular montage. The average peak EF of pre-auricular montage is 0.22 V/m, while that of infra-auricular montage is 0.13 V/m. There are significant differences in peak EF distribution for different electrode montages (pairwise *t*-test, *n* = 5, *t* = −9, *p* < 0.01). Different EF distributions were revealed when the same electrode montage was applied among participants (Figures 5B,C).

Furthermore, the EF distributions for different electrode montages by *in vivo* measurements were examined (Figures 5D,E). The measured voltage and EFs were significantly different for different electrode montages (*p* < 0.01). The prediction accuracy of the simulation model is similar for infra-auricular and pre-auricular montage (Supplementary Figure 2).

### Age Effect

To further study the age-dependent variability between adults and children, we regroup participants into two groups by their age. First, simulation results were used for comparing the CES-induced EF difference between these two groups. Then, the predictive accuracy of the simulation model was compared between two groups by combining the *in vivo* measured data.

**FIGURE 6:**
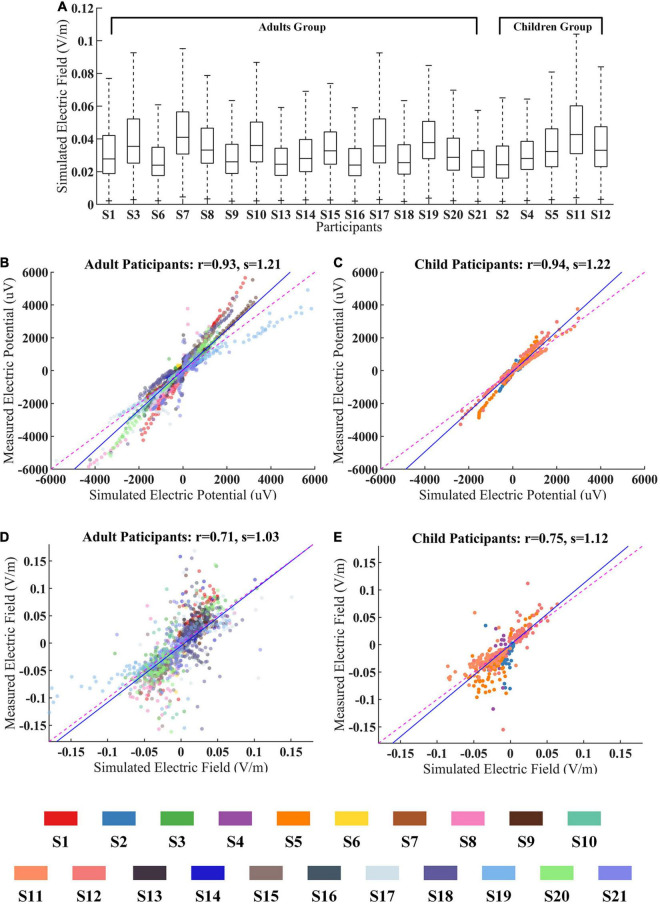
The age effect between adults and children during CES. **(A)** Simulated electric fields (EFs) for all participants within WM and GM. **(B,C)** The measured voltages and predicted ones for adults and children groups. **(D,E)** The measured EFs and predicted ones for adults and children groups. The data from different participant were represented by the color-coding of participants at the bottom of the figure.

The simulated EF distributions across adults (mean EF magnitude = 0.029 V/m) and children (mean EF magnitude = 0.036 V/m) were comparable individually (Figure 6A). We further examined the correlation between measured electric potentials and simulated ones in both groups. The recorded voltages are highly correlated with the simulated voltage values (adults group: *r* = 0.93, *p* < 0.001, *s* = 1.21; children group: *r* = 0.94, *p* < 0.001, *s* = 1.22, Figures 6B,C).

We also found that the correlation coefficients between measured EFs and simulated ones did not show significant differences for these two groups (adults group: *r* = 0.71, *p* < 0.001; children group: *r* = 0.75, *p* < 0.001, Figures 6D,E).

## Discussion

Our results demonstrate that the recorded voltages are highly correlated with the simulated values, which validated that the computational simulation model can effectively predict EF distribution during CES. This is a crucial finding for future clinical application of CES. Several previous studies have compared the tES-induced voltage changes with the simulated ones (Huang et al., 2017; Opitz et al., 2018). The validity of the simulation model is confirmed by their results. But these studies mainly focused on the EF distribution of standard tES. Although CES is a form of tES, the electrode montage is significantly different from that of standard tES. It is still unknown whether the simulation results will be affected by the different thickness of the scalp and skull in the current pathways between CES and standard tES. In addition, their *in vivo* data mainly comes from ECoG recording, the skull defect would affect the measurement results. By using minimally invasive sEEG, our study provides more accurate evidence from cortical and subcortical brain regions with larger sample size and wider electrode coverage, and confirm that the simulation model of CES is applicable and valid. On the other hand, we also observed that the slope *s* of the best linear fit is not equal to one (slope *s* = 1 represents perfect prediction). The systemic discrepancy between measured and simulated potentials may be attributable to the difference in individual conductivity. Huang et al. (2017) have tried to calibrate the models by adjusting conductivity values for each individual model with the goal of minimizing the mean square error between predicted and measured EF values. But it is worth noting that their conductivity optimization is only based on the numerical model rather than individual actual tissue conductivity. The model calibration needs to be further combined with other tissue conductivity measurements. Huang et al. (2017) also have performed a precise tissue segmentation (the skull consisted of lower compacta, spongiosa, and upper compacta) in modeling, but the prediction accuracy did not significantly improved. The anatomical fidelity and mesh resolutions are also important for accurate simulations (Saturnino et al., 2019a), especially for EF prediction in the areas of cerebral cortex folding. This suggest that future efforts to increase simulation accuracy should account for accurate anatomical representation and mesh density. To the best of our knowledge, this is the first validation study of CES modeling by directly recording the induced electric potentials with intracranial depth electrodes. The results from our study have important implications for future neuromodulation translation study and it provides a promising path to optimize CES modeling.

The level of stability of recordings over time is an important factor. According to our data from Supplementary Figure 1B, the electrical potential magnitude shows a small variation over a short time period. The average coefficient of variation of measured peak voltages is less than 1.90% within a stimulation trial (100 s). We also found that the average correlation coefficients of measured peak voltages did not show significant differences for these two groups (adults group: 2.05%; children group: 1.27%, *p* > 0.36). The observed drift in voltage magnitudes may come from several potential noise sources, such as subject movement (Rice et al., 2013), environmental noise, electrode contact and the signal amplifier. Heartbeat and respiration were also found to cause stimulation artifacts in a previous study (Noury et al., 2016).

Various electrode montages have been adopted for CES in various neurological conditions, but the effect from different electrode montages has not been well sorted out. A previous modeling study in a single subject found that CES electrode montages can change the overall brain current flow patterns (Datta et al., 2013). Consistent with a previous study (Chhatbar et al., 2018), we also observed that the CES-generated EFs highly depend on the electrode montage. As demonstrated by our study, even small electrode displacement (infra-auricular vs. pre-auricular) could incur significantly different CES-generated EFs, which may contribute to the variation in CES-induced clinical effects. We reveal, for the first time, the inter-individual differences in CES-induced EF among multiple participants in this study. Our results showed that even with the same electrode montage and the same electrical stimulation parameters, CES still induce different EF distributions among subjects. Our findings have an important translational implication and highlight the importance of a personalized approach in future CES studies.

We observed that CES-induced field intensity linearly increases with stimulation intensity. These results corroborate previous findings obtained from *in vivo* measurements of tACS (Huang et al., 2017) and tDCS (Chhatbar et al., 2018). Our results further verified these biophysical properties in WM and GM. In addition, we found that CES-induced field intensity shows small variation at different stimulation frequencies. Similar to the previous study (Huang et al., 2017), the slight voltage distortions in some recording channels were observed at various frequencies stimulation (Figures 4D,E). This may be caused by many factors, such as environmental noise and head movement. The overall variation is relatively small. The averaged coefficient of CES-induced voltage at different stimulation frequencies was less than 4.10% (Figure 4F). It is generally supposed that brain tissue would demonstrate different conductivity characteristics at the different stimulation frequencies. A previous CES simulation study reported a significant difference in peak EF between DC and AC (150 Hz) stimulation in the same montage where different conductive values are adopted in DC and AC simulation (Datta et al., 2013). However, when the measured CES-induced potentials are compared between 0.5 and 200 Hz stimulation at the same electrode, they are almost the same across all examined electrodes. Our results suggest that the conductivity characteristics of brain tissues keep stable under the CES with different frequencies from 0.5 to 200 Hz. Based on the intensity and frequency characteristics, we can speculate that the brain could be considered a resistive conductor under a low-frequency electrical stimulation. The propagative, inductive, and capacitive effects can be negligible. Therefore, the theoretical basis of modeling is suitable.

Because of the lack of direct *in vivo* evidence for CES-induced EF distributions, it is still controversial whether CES can provide sufficient intracerebral currents and influence neural activity. Our clinical sEEG recordings indicate that the maximal EF magnitude could reach 0.4 V/m (peak value) for the 1.0 mA CES. Generally, the maximum output current value is less than 500 μA for most CES devices. The induced EF magnitude is less than 1.0 V/m level, which was likely required to generate neuromodulatory effects (Vöröslakos et al., 2018). However, we notice that the 1.0 V/m concept was derived from patch-clamp recordings in rodent cortex *in vivo*. It is not clear how these findings can be extrapolated to *in vivo* physiological effects in humans. There was also a published report that 0.2 V/m was found to be adequate to entrain coherent gamma oscillations *in vitro* (Reato et al., 2010).

Our study provided new insight into the impact of age on CES. The skull and brain gradually mature as children grow, and it likely influences the dielectric properties of tissue (Minhas et al., 2012). Although accurate models require individual tissue conductivity values, only a few studies reported the conductivity values for children (McCann et al., 2019). Consistent with several previous studies (Minhas et al., 2012; Kessler et al., 2013; Ciechanski et al., 2018), the tissue conductivity values commonly used in adults were applied to the pediatric modeling in this study. Interestingly, the prediction accuracy of the simulation model is similar between adults and children. It hints that the conductivity values used in adults are likely applicable in children. Additionally, there are apparent differences in skull thickness, CSF and brain volumes between adults and children (Group, 2012). Variations in head anatomy have an important influence on the strength and distribution of EFs in humans. We also observed inter-individual variability among participants. Generally, the head size and tissue thickness were greater in adults than in the children (Antonenko et al., 2021a). A larger head size may be associated with a lower EF strength across montages from simulation results (Antonenko et al., 2021a). Consistent with several previous studies (Minhas et al., 2012; Kessler et al., 2013), we also found on average, children will be exposed to higher EFs than adults when the same current intensity is applied (Figure 6A). The high correlation between the measured EFs and simulated ones suggested that the personalized model has taken the anatomic difference into account when individualizing computational simulation.

Our study is not free from limitations. First, precise tissue segmentation and individualized conductivity are the future directions and bring further simulation optimization. Our simulation head model was only segmented into several common brain tissue components (WM, GM, CSF, skull, and scalp). It would be desirable to include more subdivided tissue components. Second, the tissue conductivities adopted in our study are widely used (Huang et al., 2019), but the accuracy could be further optimized as many studies suggested. Third, several studies suggest employing the anisotropic simulation model for optimization (Shahid et al., 2014; Metwally et al., 2015). However, we could not adopt the anisotropic simulation model due to the lack of DTI data. We also notice that a recent study suggested that WM electrical conductivity could be isotropic (Koessler et al., 2017). Huang et al. (2017) also reported that the prediction performance of the simulation model does not be improved by adding anisotropic WM. Further work needs to determine additive benefit from anisotropic modeling. Fourth, SimNIBS is also a well-known and widely used tool for simulating the EF distributions of tES (Alekseichuk et al., 2019; Saturnino et al., 2019b). It is significant to compare the EF predictions by using different modeling pipelines (Puonti et al., 2020; Bhalerao et al., 2021). Fifth, Multi-electrode tES, such as dual site tACS (Alekseichuk et al., 2019), is a promising technique for modulating brain oscillations. Studying the multi-electrode tES-induced EFs by *in vivo* measurements is of great significance in future clinical studies. Lastly, it is not easy to conduct such a study on this special diseased patient population. Although we recruited 21 participants including 5 children, the sample size of child participants is relatively small as compared to the other studies (Ciechanski et al., 2018; Antonenko et al., 2021b).

## Conclusion

Our *in vivo* measurements demonstrate that the individualized simulation model can reliably predict the CES-induced EFs in both adults and children. It also confirms that the CES-induced EFs highly depend on the electrode montages and individual anatomical features. Our findings have important translational implications in brain modulation, and it highlights the importance of a personalized approach in future CES studies.

## Data Availability Statement

The raw data supporting the conclusions of this article will be made available upon reasonable request.

## Ethics Statement

The studies involving human participants were reviewed and approved by The Institutional Review Board at the Second Affiliated Hospital Zhejiang University School of Medicine. Written informed consent to participate in this study was provided by the participants’ legal guardian/next of kin.

## Author Contributions

MW: study conceptualization, methodology, software, formal analysis, investigation, writing – original draft, and visualization. TF: investigation and writing – original draft. HJ: validation, formal analysis, and investigation. JuZ and WF: study conceptualization and writing – original draft. PC: formal analysis and writing – original draft. JiZ: study conceptualization, resources, supervision, and funding acquisition. SZ: study conceptualization, writing – original draft, resources, supervision, and funding acquisition. All authors contributed to the article and approved the submitted version.

## Conflict of Interest

The authors declare that the research was conducted in the absence of any commercial or financial relationships that could be construed as a potential conflict of interest.

## Publisher’s Note

All claims expressed in this article are solely those of the authors and do not necessarily represent those of their affiliated organizations, or those of the publisher, the editors and the reviewers. Any product that may be evaluated in this article, or claim that may be made by its manufacturer, is not guaranteed or endorsed by the publisher.

## Footnotes

1
https://neuroimage.usc.edu/brainstorm/

2
https://www.parralab.org/roast/
