# Supplementary material for: In vivo Measurements of Electric Fields During Cranial Electrical Stimulation in the Human Brain
Source: Front Hum Neurosci. 2022 Feb 18;16:829745. doi: 10.3389/fnhum.2022.829745 (PMC8895368; doi:10.3389/fnhum.2022.829745)
Supplement: Supplementary file 1 [file Data_Sheet_1.PDF]

Supplementary Material

Supplementary Table I. Implementation of different stimulus pattern for participants. Due to the restrains in our clinical trials, not all participants receive all stimulation studies.

Participants	Group	Various Intensities	Various Frequencies	Infra-auricular Montage	Pre-auricular Montage
S1	Adult	√		√	
S2	child	√	√	√	
S3	Adult	√	√	√	
S4	child	√	√	√	
S5	child	√	√	√	
S6	Adult			√	√
S7	Adult			√	√
S8	Adult		√	√	√
S9	Adult			√	√
S10	Adult			√	√
S11	child			√	
S12	child			√	
S13	Adult			√	
S14	Adult			√	
S15	Adult			√	
S16	Adult			√	
S17	Adult			√	
S18	Adult			√	
S19	Adult			√	
S20	Adult			√	
S21	Adult			√	

√ represent participant received this type of stimulation process.

Supplementary Table II. The correlation coefficients and linear fit-slope among participants

	Voltage		Projected EFs	
**Participants**	***r***	***s***	***r***	***s***
S1	0.99	1.96	0.913	1.97
S2	0.94	1.38	0.83	1.24
S3	0.977	1.08	0.78	0.97
S4	0.937	1.29	0.662	1.02
S5	0.98	1.55	0.808	1.38
S6	0.83	1.09	0.56	2.12
S7	0.98	1.03	0.801	1.43
S8	0.97	1.51	0.77	1.03
S9	0.978	1.28	0.791	1.47
S10	0.94	1.21	0.82	1.13
S11	0.98	0.997	0.76	1.15
S12	0.98	1.29	0.72	1.214
S13	0.89	1.93	0.58	1.32
S14	0.93	0.76	0.69	0.82
S15	0.98	1.73	0.65	1.31
S16	0.96	1.32	0.57	1.43
S17	0.90	1.07	0.53	0.98
S18	0.96	1.53	0.59	0.76
S19	0.97	0.74	0.77	0.68
S20	0.99	1.51	0.87	1.52
S21	0.84	1.56	0.49	1.31

r, s represent correlation coefficients and slope of a linear fit between measured and simulated values, respectively.

Supplementary Figure 1

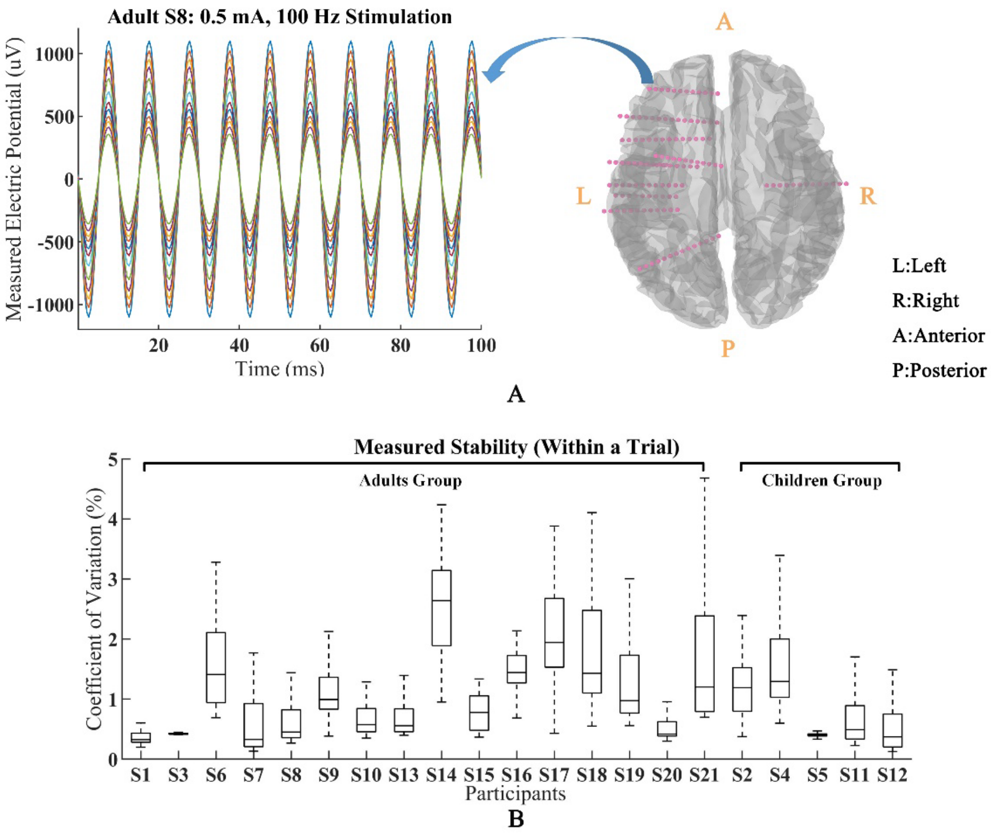

Supplementary Figure 1 Intracranial voltage recordings and measured stability. (**A**) The left figure is an example of filtered recording signals from participant S8. Each curve represents a different electrode. (**B**) Under 100 Hz/500 μA CES, the coefficient of variation of measured peak voltages within a stimulation trial. The average correlation coefficients did not show significant differences for these two groups (adults group: 2.05%; children group: 1.27%, $p > 0.36$). *p-values* were derived from two-sided Mann-Whitney U tests.

Supplementary Figure 2

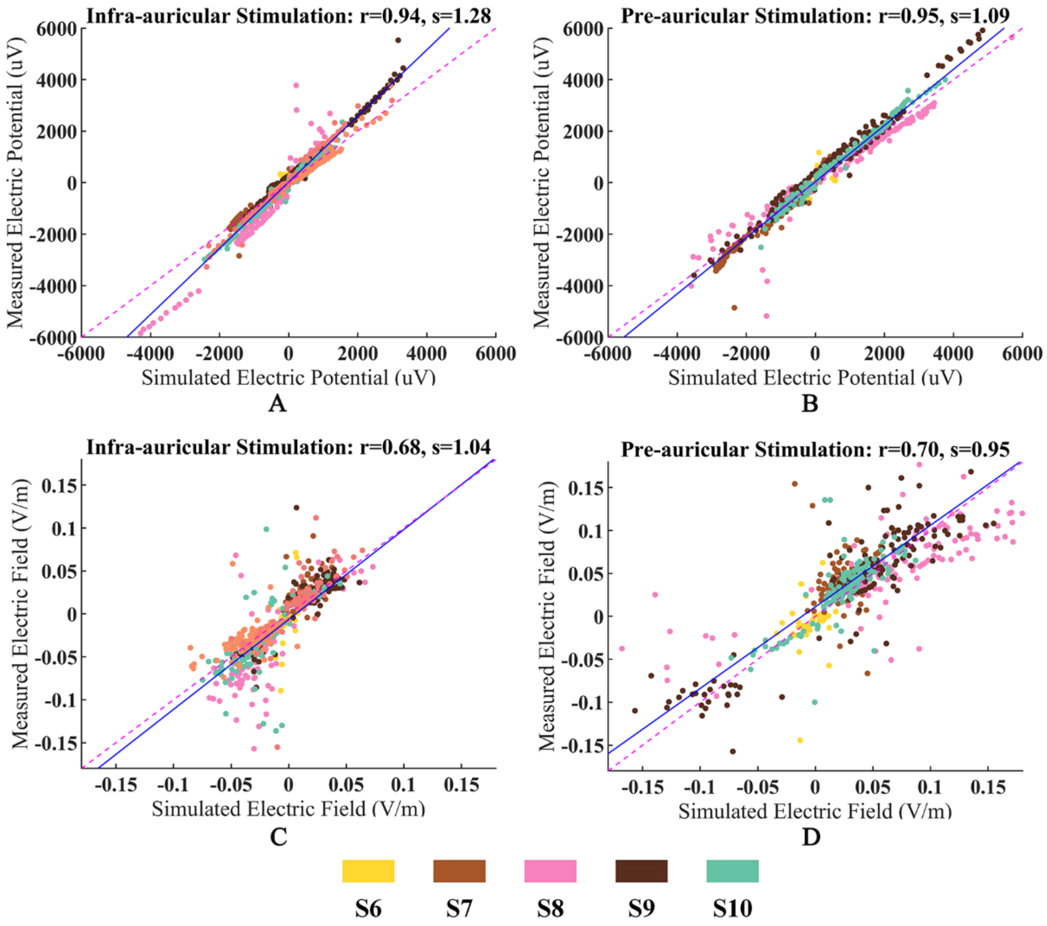

Supplementary Figure 2 Correlation between simulated and measured values for infra-auricular and pre-auricular montage. The data from different participant were represented by the color-coding of participants at the bottom of the figure. Points falling on the magenta line represent perfect prediction (slope $s = 1$). Blue line represents fitting line.

Supplementary Figure 3

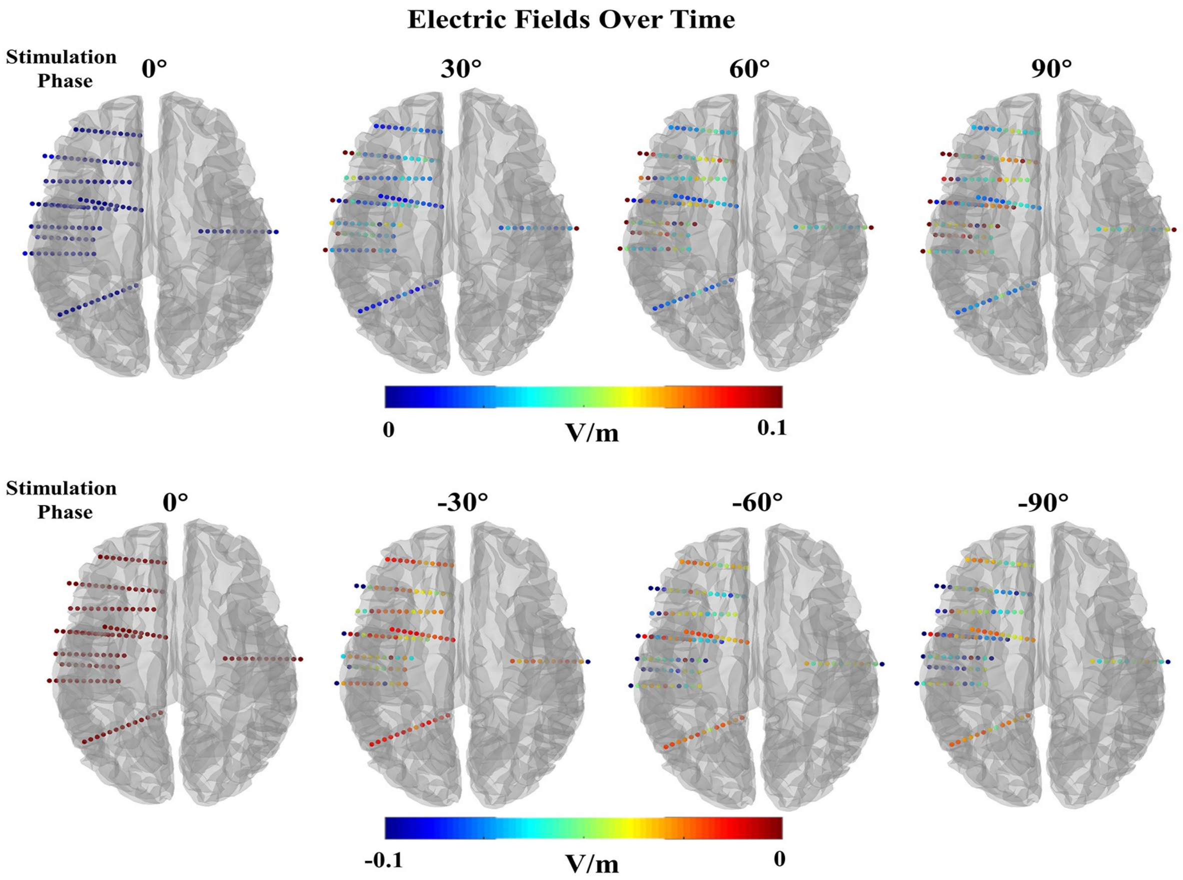

Supplementary Figure 3 During 100 Hz/500 μA CES, the electric fields in the brain over time for participant S8. The color encodes the electric field magnitude.
